# *NPHS2 *variation in focal and segmental glomerulosclerosis

**DOI:** 10.1186/1471-2369-9-13

**Published:** 2008-09-29

**Authors:** Stephen J Tonna, Alexander Needham, Krishna Polu, Andrea Uscinski, Gerald B Appel, Ronald J Falk, Avi Katz, Salah Al-Waheeb, Bernard S Kaplan, George Jerums, Judy Savige, Jennifer Harmon, Kang Zhang, Gary C Curhan, Martin R Pollak

**Affiliations:** 1 Renal Division, Department of Medicine, Brigham and Women's Hospital, Boston, Massachusetts, USA; 2 Glomerular Disease Center, Columbia University College of Physicians and Surgeons, New York, NY, USA; 3 Division of Nephrology and Hypertension, Department of Medicine, UNC Kidney Center, University Of North Carolina, Chapel Hill, North Carolina, USA; 4 Department of Pediatrics, University of Alabama, Birmingham, Alabama, USA; 5 Department of Pediatrics, Division of Nephrology, The Children's Hospital of Philadelphia, Philadelphia, PA, USA; 6 Endocrine Centre and Department of Medicine, Heidelberg Repatriation Hospital, Victoria, Australia; 7 Department of Medicine, The Northern Hospital, Epping, Victoria, Australia; 8 Johan A. Moran Eye Center and Department of Ophthalmology & Visual Science, University of Utah, Salt Lake City, USA; 9 Channing Laboratory, Department of Medicine, Brigham and Women's Hospital, Boston, Massachusetts, USA

## Abstract

**Background:**

Focal and segmental glomerulosclerosis (FSGS) is the most common histologic pattern of renal injury seen in adults with idiopathic proteinuria. Homozygous or compound heterozygous mutations in the podocin gene *NPHS2 *are found in 10–30% of pediatric cases of steroid resistant nephrosis and/or FSGS.

**Methods:**

We studied the spectrum of genetic variation in 371 individuals with predominantly late onset FSGS (mean age of onset 25 years) by analysis of DNA samples.

**Results:**

We identified 15 non-synonymous alleles that changed the amino acid sequence in 63 of the subjects screened (17%). Eight of these (p.R138Q, p.V180M, p.R229Q, p.E237Q, p.A242V, p.A284V, p.L327F and the frameshift 855–856 delAA) are alleles previously reported to cause FSGS in either the homozygous or compound heterozygous states, while the remaining 7 (p.R10T, p.V127W, p.Q215X, p.T232I, p.L270F, p.L312V and the frameshift 397delA) are novel alleles that have not been demonstrated previously. Twelve individuals of the 371 (3.2%) screened had two likely disease-causing *NPHS2 *alleles, present in either a homozygous or compound heterozygous state. We genotyped the two most common of the non-synonymous *NPHS2 *alleles (p.A242V and p.R229Q) identified by resequencing in participants from the Nurses' Health Study and also genotyped p.R229Q in 3 diabetic cohorts. We found that the presence of either of these variants does not significantly alter the risk of albuminuria in the Nurses' Health participants, nor does p.R229Q associate with "diabetic nephropathy".

**Conclusion:**

*NPHS2 *mutations are a rare cause of FSGS in adults. The most common non-synonymous *NPHS2 *variants, p.R229Q and p.A242V, do not appear to alter the risk of proteinuria in the general population nor does p.R229Q associate with measures of kidney dysfunction in diabetic individuals. Our results help clarify the frequency of FSGS-causing *NPHS2 *mutations in adults and broaden our understanding of the spectrum of *NPHS2 *mutations that lead to human disease.

## Background

Focal and segmental glomerulosclerosis (FSGS) is now the most common histologic pattern of injury seen in adults with primary glomerular disease [1]. Rather than a single disease entity, FSGS describes a pattern of injury seen in kidney damage secondary to a number of identifiable primary causes but also seen as an idiopathic, isolated finding. Over the past decade, human genetic studies have confirmed the heterogeneity of the underlying biological cause of this histologic pattern. Heterozygosity for mutations in *ACTN4*, *TRPC6*, and *CD2AP *cause rare forms of steroid resistant FSGS [2-5]. Mutations in both *NPHS2 *(podocin) alleles also cause steroid resistant FSGS [6] and are a much more common cause of FSGS than mutations in other genes identified to date [7]. Podocin mutations appear to cause 10–30% of childhood steroid resistant nephrotic syndrome [8]. Although genetically distinct forms of FSGS may follow different patterns of inheritance, the pattern can be difficult to identify, particularly in small families.

The clinical utility of genetic testing in the evaluation of FSGS and nephrotic syndrome in adults remains unclear. He et al found disease-segregating *NPHS2 *mutations in only 1 of 87 FSGS subjects analyzed [9]. McKenzie et al have suggested that homozygous or compound heterozygous mutations in *NPHS2 *are very rare causes of sporadic, adult onset FSGS. They also found that heterozygotes for R138Q are more common in cases with FSGS than controls without the disease and reported that a common haplotype in *NPHS2 *modifies disease risk in African Americans but not European Americans [10].

Kidney biopsies are generally performed late in the evaluation of a nephrotic child who does not respond to steroids. Early identification of unambiguous disease-causing mutations in both *NPHS2 *alleles could lead to avoidance of prolonged glucocorticoid therapy and perhaps the need for kidney biopsy in these patients. The clinical utility of *NPHS2 *mutation analysis is much less clear in adults as there exists a broader span of underlying etiologies in the differential diagnosis in this age group and the histologic lesions underlying nephrotic and subnephrotic proteinuria overlap considerably. Here, we performed mutational analysis of *NPHS2 *in a large group of probands with FSGS to define the contribution of *NPHS2 *to late-onset disease. In addition, we genotyped two relatively common non-synonymous variants (cSNPs) in several sample sets to assess the possible contribution of these variants to albuminuria in both the general adult population and among diabetics, a group at high risk for the development of proteinuric kidney disease.

## Methods

### Patients

Three hundred and seventy-one unrelated individuals diagnosed with FSGS were studied. Of these, 122 (33%) had at least one affected relative, while the remaining 249 (67%) were sporadic cases without a family history. Most of these samples were ascertained after referral by a nephrologist caring for the subject and one or more family members.

Two hundred (54%) of the subjects were Caucasian. The remaining 171 were either African American (n = 68, 18.2%), Hispanic (n = 26, 7%), Asian (n = 6, 1.6%), American Indian (n = 1, 0.2%) or of unreported ethnicity (n = 70, 19%). Families and sporadic cases with radiologic, clinical or histopathologic findings consistent with secondary forms of FSGS were excluded from the analysis. The median age of onset of disease in the subjects studied was 25 years (range 0.25-to-65 years of age). The median age of onset of FSGS in the familial cases was 16 years (range 1.20-to 50 years of age), while that of sporadic patients was 26 years (range 1.25-to-69 years).

### Nurses' Health study participants and type 1 and type 2 diabetic patients

We genotyped samples from women enrolled in the Nurses' Health Study I or II [11]. The Nurses' Health Study (NHS) samples are large prospective cohorts of female registered nurses. We also genotyped 1988 samples from three distinct study groups, the University of Utah diabetes study, the Genetics of Kidney Disease in Diabetes study (GoKinD), and Australian endocrine diabetics. University of Utah diabetes study: n = 280; 41 type 1 diabetic individuals and 239 with type 2 diabetes. Twenty patients (49%) with type 1 diabetes and 89 patients (37%) with type 2 diabetes had end-stage renal disease. Diabetic patients without end-stage renal disease were used as controls (21/41, 51% of type 1 diabetics; 150/239, 63% of type 2 diabetics). Median age of type 2 diabetic cases was 22 years, and controls with type 2 diabetes was 19 years. The median age of the type 1 diabetic cases and controls was not available). Genetics of Kidney Disease in Diabetes (GoKinD) study group: n = 1,279; 455 "case" samples have end-stage renal disease and 824 who do not have end-stage renal disease were used as "control" subjects. All GoKinD subjects have long-standing (10+ years) of type 1 diabetes. Median age of the cases was 44, and median age of controls was 40 years. Australian endocrine diabetes group: n = 429; 67 type 1 diabetic subjects and 362 with type 2 disease. Fifty-one of the type 1 diabetics had an urinary albumin excretion rate (AER) <20 μg/min, 10 had an AER between 20 μg/min and 200 μg/min, and the remaining 6 had an AER >20 μg/min). Two hundred and twenty-eight of the type 2 diabetics had an AER<20 μg/min, 83 had an AER between 20 μg/min and 200 μg/min, and the remaining 46 had an AER >20 μg/min. The median age of the type 1 microalbuminurics was 55 years, that of macroalbuminurics was also 55 years and normoalbuminurics was 53 years. The median age of the type 2 diabetics with microalbuminuria was 67 years, that of macroalbuminurics was 68 years and normoalbuminurics was 70 years.

### Informed Consent

Studies were performed in accordance with human subject protocols approved by the human research committees at each of the institutions.

### Genetic analyses

#### NPHS2 sequence analysis

Genomic DNA was extracted from peripheral blood cells using the QIAmp DNA blood kit (QIAGEN Inc., Valencia, California, USA). Total genomic DNA (20–25 ng) was amplified using primers designed from the analysis of the available genomic sequence (*Homo sapiens *chromosome 1 BAC clone RP11-545A16, GeneBank accession number AL160286). An ABI 3730xl DNA analyzer was used for sequence analysis. Primers used for sequence analysis are available on request.

#### Mutation validation

Novel variants that were not identified in previous studies of *NPHS2 *were studied for their frequency in a cohort of 362 non-diseased Caucasian HapMap CEPH control alleles and, when possible, co-segregation with disease in the respective families. In both instances, genotyping was performed using MALDI-TOF mass spectroscopy based SNP genotyping (Sequenom) at the Harvard Partners Genotyping Facility. Large control cohorts with no known kidney disease from other ethnic groups were not necessary in this study, as most of the patients in whom we identified novel mutations were Caucasian. Only one sample was non-Caucasian, and control samples from this ethnic group (Sri-Lanken) was not available.

#### Genotyping of p.R229Q and p.A242V

One thousand nine hundred and eighty eight diabetic samples from 3 distinct study groups with either type 1 or type 2 diabetes were genotyped for the p.R229Q variant using either an p.R229Q TaqMan allelic discrimination assay, the MALDI-TOF mass spectroscopy Sequenom based SNP genotyping at the Harvard Partners Genotyping Facility or *Cla I *digestion of exon 5 *NPHS2 *amplicons.

We designed a TaqMan allelic discrimination assay that used a specific fluorescent, dye-labeled probe for both the wild type (G755G) and mutant p.R229Q (G755A) alleles. The sequences of the probes and primers were: wildtype probe (Allele G) AGGGATCGATGTGCT-VIC dye at the 5' end and MGB quencher at the 3' end, mutant probe (Allele A) TGAGGGATTGATGTGC-FAM at the 5' end and MGB quencher at the 3' end, forward primer AATTCCTTGTGCAAACCACTATGAA, reverse primer CGATGCTCTTCCTCTCTAGAAGAATTT. A 25 μL reaction was prepared for each sample analyzed, containing 12.5 μL of TaqMan Universal PCR Master Mix containing AmpliTaq Gold DNA polymerase and other reagents (Applied Biosystems, Foster City, CA, USA), 9.5 μL of DNase, RNase and Protease free Molecular grade water (Cellgro, Lawrence, KS, USA), 0.5 μL of 100 pmole concentration of forward and reverse primers (Applied Biosystems, Foster City, CA, USA), 0.0625 μL of 100 uM concentration of Allele G and Allele A Taqman MGB probes (Applied Biosystems, Foster City, CA, USA) and 2.5 μL of 25 ng total genomic DNA or p.R229Q plasmid DNA. Controls for this assay consisted of non-template controls (NTC), 3 FSGS patients and 1 sibling from each of these subjects, that either have or were not shown to have p.R229Q in a heterozygous state from prior studies respectively [6]), and an p.R229Q plasmid (homozygous control). Genotyping was performed in an Applied Biosystems 7300/7500 Real-Time PCR machine (Applied Biosystems, Foster City, CA, USA).

All 429 samples from the Australian endocrine diabetics were examined for p.R229Q using *Cla1 *digestion [6] and further verified using direct sequencing performed with the ABI Prism Big Dye Terminator Cycle Sequencing-ready reaction kit (PE Applied Biosystems) by the Australian Genome Research Facility using a Perkin-Elmer 377 automated sequencer.

#### Nurses' Health Study Group

Samples from either the Nurses' Health Study I or II were genotyped for p.A242V or p.R229Q using a MALDI-TOF mass spectroscopy Sequenom based SNP genotyping assay developed and performed at the Harvard Partners Genotyping Facility. Four Caucasian controls (CEPH) samples from the international HapMap project without at least one p.R229Q or p.A242V allele were genotyped at the same time as the patient samples, and these were consistently negative for both the c.686G>A and c.725C>T of p.R229Q and p.A242V respectively. These variants do not exist in public SNP databases nor are they present on current Affymetrix genotyping SNP panels to our knowledge.

### Statistics

The frequency distributions of alleles was assessed using StatView for Windows, version 5.0 and SAS version 9.0.

## Results

### Sequence analysis of *NPHS2*: non-synonymous variants

We directly sequenced the entire coding region of the *NPHS2 *gene in PCR amplified DNA from 371 individuals, 122 of whom also had at least 1 relative with FSGS. We identified fifteen alleles that changed the predicted amino acid sequence in 63 patients (or 17% of the samples screened). These were either missense (p.R10T (c.29G>C), p.V127W (c.379G>T), p.R138Q (c.413G>A), p.V180M (c.538G>A), p.R229Q (c.686G>A), p.T232I (c.694C>T), p.E237Q (c.709G>C), p.A242V (c.725 C>T), p.L270F (c.810G>T), p.A284V (c.851C>T), p.L312V (c.934C>G) and p.L327F (c.1048C>T), truncation (p.Q215X (c.643C>T) or frameshift variants (397delA and 855-856delAA) (Table 1). Twelve of these 63 patients had non-synonymous variants in two alleles; 3 were homozygous for p.V127W, p.R138Q or p.V180M, and the remaining 9 were compound heterozygous for p.R138Q and/or p.R229Q and a variety of other alleles (Table 2).

**Table 1:** Non-synonymous *NPHS2 *variants detected

Type of variant	Nucleotide change	Effect on coding sequence	Exon	Heterozygous (n, %)	Homozygous (n, %)	Frequency in Familial FSGS	Frequency in Sporadic FSGS
missense	c.29G>C	p.R10T	1	1, 0.27%	-	N = 0	N = 1 (1/249, 0.4%)
	c.379G>T	p.V127W	2	-	1, 0.27%	N = 0	N = 1 (1/249, 0.4%)
	c.413G>A	p.R138Q	3	5, 1.3%	1, 0.27%	N = 5 (5/122, 4%)	N = 1 (1/249, 0.4%)
	c.538G>A	p.V180M	5	-	1, 0.27%	N = 1 (1/122, 0.8%)	N = 0
	c.643C>T	p.Q215X	5	1, 0.27%	-	N = 1 (1/122, 0.8%)	N = 0
	c.686G>A	p.R229Q	5	40, 10.8%	1, 0.27%	N = 10 (10/122, 8.2%)	N = 31 (31/249, 12.5%)
	c.694C>T	p.T232I	5	1, 0.27%	-	N = 0	N = 1 (1/249, 0.4%)
	c.709G>C	p.E237Q	5	1, 0.27%	-	N = 0	N = 1 (1/249, 0.4%)
	c.725C>T	p.A242V	5	14, 3.7%	1, 0.27%	N = 1 (1/122, 0.8%)	N = 14 (14/249, 5.6%)
	c.810G>T	p.L270F	6	1, 0.27%	-	N = 0	N = 1 (1/249, 0.4%)
	c.851C>T	p.A284V	7	2, 0.5%	1, 0.27%	N = 0	N = 3 (3/249, 1.2%)
	c.934C>G	p.L312V	8	1, 0.27%	-	N = 1 (1/122, 0.8%)	N = 0
	c.1048C>T	p.L327F	8	1, 0.27%	-	N = 0	N = 1 (1/249, 0.4%)
Frame-shift	397delA	Frame-shift	3	1, 0.27%	-	N = 0	N = 1 (1/249, 0.4%)
	855/6delAA	Frame-shift	7	1, 0.27%	-	N = 0	N = 1 (1/249, 0.4%)

**Table 2:** Clinical characteristics of patients with homozygous and compound heterozygous non-synonymous *NPHS2 *variants

Proband screened	Ethnicity	Homozygous or Compound heterozygous	Previously published as disease causing	Affected family members	Age of onset (years)	Response to immunosuppressive treatment	Tx/ Recurrence
FG-HU-11^S^	Sri-Lanken	p.V127W/p.V127W	No	FG-HU-11	4	NA	NA
FG-FW-12 F	Caucasian	p.R138Q/p.R138Q	[8,12-15,17,18]	FG-FW-12 FG-FW-11 FG-FW-13 FG-FW-14	4 8 4 2	No No No No	Yes/No Yes/No Yes/No No
FG-HN-11^F^	Caucasian	p.R138Q/p.Q215X	No	FG-HN-11 FG-HN-111	8 1.08	No No	Yes/No No
FG-EJ-2112^F^	Caucasian	p.R138Q/p.R229Q	[6]	FG-EJ-2112 FG-EJ-2115 FG-EJ-2116	5 3 3	NA No No	No Yes/Yes Yes/Yes
FG-IV-11^F^*	Caucasian/Lebanese	p.V180M/p.V180M	[12]	FG-IV-11 FG-IV-12	14 17	Partial NA	No NA
UNC-530^S^	NA	p.R229Q/p.R10T	No	UNC-530	18	NA	NA
CPMC-93^S^	Caucasian	p.R229Q/p.L270F	No	CPMC-93	38	Partial	NA
FG-HP-11^S^	Hispanic	p.R229Q/p.A284V	[8,15,17,19]	FG-HP-11 FG-HP-12	17 NA	No No	No Yes/No
CPMC-2^S^	Hispanic	p.R229Q/p.A284V	[8,15,17,19]	CPMC-2	21	NA	NA
CPMC-6^S^	Caucasian	p.R229Q/p.A284V	[8,15,17,19]	CPMC-6	27	NA	NA
ST-11^S^	Caucasian	p.R229Q/p.L327F	[6]	ST-11	3	No	NA
CPMC-28^S^	Caucasian	397delA/855/6delAA	No	CPMC-28	27	No	NA

F; individual with at least one other affected family member with FSGS or proteinuria, F*; individual with at least one other affected family member from a consanguineous marriage with FSGS or proteinuria, S; patient without a family history of FSGS NA; data not available.

### Homozygous non-synonymous and compound heterozygous variants

In 3 of the 122 families studied (2.5%), the p.R138Q, p.V127W and p.V180M alleles were demonstrated in a homozygous state (Table 2). In families with multiple affected individuals available for genetic analysis, these alleles segregated in a pattern consistent with autosomal recessive transmission. We identified 9 compound heterozygous events. A majority (7/9 or 78%) of these consisted of p.R229Q, inherited together either with a rarer allele (n = 4), p.R138Q (n = 2) or p.A284V (n = 3). These mutations were identified in 2 index cases with a family history of disease and 7 sporadic cases (Table 2). In the proband's family, the alleles segregated with disease in an autosomal recessive manner. In addition, we identified p.R229Q and p.A242V in homozygous and compound heterozygous states. The p.R229Q and p.A242V homozygous events were identified in two unrelated sporadic patients (CPMC-96 and FG-GC-1112 respectively), and the p.R229Q/p.A242V compound heterozygous state was found in another unrelated sporadic patient (CH-1).

The p.R10T, p.V127W, p.Q215X and p.L270F alleles (present in a compound heterozygous state with either p.R138Q or p.R229Q (Table 2)) were not observed in 362 non-diseased control alleles. Only 1 proband with 2 deleterious *NPHS2 *alleles other than p.R229Q developed disease in adulthood (Table 2). The 4 other probands with adult onset disease and significant *NPHS2 *variants in both alleles had one p.R229Q variant. This supports the notion that in the absence of a p.R229Q *NPHS2 *variant, *NPHS2 *defects are unlikely to be the cause of disease in an adult with FSGS undergoing genetic analysis.

### Single heterozygous alleles

Fifty-one patients had a single heterozygous *NPHS2 *allele predicted to alter the encoded protein. These variants (and the respective number of patients they were demonstrated in) were: p.R138Q (n = 3), p.R229Q (n = 32), p. T232I (n = 1), p.E237Q (n = 1), p.A242V (n = 13) and p.L312V (n = 1). Both the p.T232I (c.694C>T) and p.L312V (c.934C>G) alleles were absent in 362 non-diseased control alleles sequenced, while all the others have been demonstrated by previous studies [6-8,12-24]. The p.T232I variant was identified in an 8 year-old African-American boy (UAB-023) with no family history who developed FSGS at the age of 2.5 years. The p.L312V mutation was identified in 2 of 4 siblings in a heterozygous state, both of whom had biopsy-confirmed FSGS (Family FG-DK). No second mutant allele was found. Both parents were deceased, the father from kidney failure secondary to prostrate cancer and the mother for unknown reasons.

### Genotyping of p.R229Q and p.A242V in women enrolled in the Nurses' Health Study

The p.R229Q and p.A242V variants were the most common non-synonymous variants demonstrated in our FSGS patient cohort (with allele frequencies of 0.02 and 0.06 respectively). The frequency of p.R229Q did not differ between the FSGS races studied, whereas the p.A242V variant was present in a higher proportion of African Americans with FSGS compared to other races (chi-square with Yates correction 9.79, p = 0.00057). We were interested in whether both the p.R229Q and p.A242V variants might contribute to the risk of renal impairment in the general population by investigating whether these variants associate with increased urinary albumin/creatinine ratio, an early marker of renal disease.

We genotyped the p.R229Q variant in a cohort of 2,596 women aged over 50 years who were enrolled in 1976 to participate in the Nurses' Health Study I (of which 97% were of Western european descent). We genotyped the p.A242V variant in 1559 participants in the Nurses' Health Study II (age 44 or greater; 94% of Western european descent). The allele frequencies of the p.R229Q and p.A242V variants in these cohorts were 0.0352 and 0.034, respectively. The genotype frequencies of p.R229Q and p.A242V heterozygotes were 6.5% and 7.0% respectively. Homozygous events were rare for both variants, with genotype frequencies of 0.1% and 0.26% (for p.R229Q and p.A242V respectively). We observed no association between the presence of p.R229Q and p.A242V in either of the homozygous or heterozygous states with increased urinary albumin/creatinine ratio.

### Association studies of p.R229Q with diabetic nephropathy

We were also interested in investigating whether p.R229Q might represent a modifying allele for renal disease in a more common cause of renal impairment, diabetic nephropathy. The p.R229Q variant has been associated with microalbuminuric events (designated by semi quantitative protocol) in an urban population isolated from Brazil [25] and has shown to cause FSGS in the compound heterozygous state along with rarer non-synonymous variants [6]. We genotyped p.R229Q in 1988 diabetic patients from 3 different cohorts and investigated for associations between p.R229Q with end-stage renal disease and proteinuria (Table 3).

**Table 3:** End-stage renal disease and albuminuria associations with p.R229Q in different diabetic cohorts

	N = 280	Type I (n = 41)		Type 2 (n = 239)		Total (n = 280)	
Utah diabetes study	No ESRD (n = 171)	21 p.R229R	0 p.R229Q	140 p.R229R	10 p.R229Q	161 p.R229R	10 p.R229Q
	ESRD (n = 109)	17 p.R229R	3 p.R229Q (χ^2 ^3.40, p = 0.07)	82 p.R229R	7 p.R229Q (2%) (χ^2 ^0.12, p = 0.73)	99 p.R229R	10 p.R229Q (2%) (χ^2 ^1.11, p = 0.29)
	N = 1,279	Type I (n = 1,279)		Type 2 -		Total -	
GoKinD	No ESRD (n = 824)	759 p.R229R	65 p.R229Q	-	-	-	-
	ESRD (n = 455)	428 p.R229R	p.27 R229Q (χ^2 ^1.68, p = 0.20)	-	-	-	-
	N = 429	Type I (n = 67)		Type 2 (n = 357)		Total (n = 429)	
Australian endocrine	Normo (n = 279)	47 p.R229R	4 p.R229Q	206 p.R229R	22 p.R229Q	253 p.R229R	26 p.R229Q
	Micro (n = 93)	8 p.R229R	2 p.R229Q (χ^2 ^1.39, p = 0.24)	77 p.R229R	6 p.R229Q (2%) (χ^2 ^0.44, p = 0.51)	85 p.R229R	8 p.R229Q (2%) (χ^2 ^0.04, p = 0.84)
	Macro (n = 52)	6 p.R229R	0 p.R229Q (χ^2 ^0.5, p = 0.48)	42 p.R229R	4 p.R229Q (2%) (χ^2 ^0.04, p = 0.84)	48 p.R229R	4 p.R229Q (2%) (χ^2 ^0.14, p = 0.71)
	Micro- or Macro (n = 145)	14 p.R229R	2 p.R229Q (χ^2 ^0.32, p = 0.57)	119 p.R229R	10 p.R229Q (2%) (χ^2 ^0.36, p = 0.55)	133 p.R229R	12 p.R229Q (2%) (χ^2 ^0.13, p = 0.72)

The frequency of p.R229Q in patients with type 1 or type 2 diabetes with end-stage renal disease (ESRD) was compared to those without for the Utah diabetes study and Genetics of Kidney Disease in Diabetes (GoKinD) participants. Likewise, the frequency of p.R229Q in patients with microalbuminuria (Micro) or macroalbuminuria (Macro) was compared with the frequency of normoalbuminuria (Normo) in patients with type 1 or type 2 diabetes in the Australian endocrine patients. No associations were found.

#### Utah diabetics

Two hundred and eighty patients with either type 1 diabetes (n = 41, 15%) or type 2 diabetes (n = 239, 85%) were studied. Twenty patients (49%) with type 1 diabetes and 89 patients (37%) with type 2 diabetes had end-stage renal disease. Diabetic patients without end-stage renal disease were used as controls (21/41, 51% of type 1 diabetics; 150/239, 63% of type 2 diabetics). The allele frequency of R229Q in the diabetic cases with end-stage renal disease was 0.036, and 0.030 in the controls without ESRD (Table 3). No associations between p.R229Q and end-stage renal disease in either type 1 or type 2 diabetes were found (chi-square 3.39 and 0.121 with p values of 0.07 and 0.73 respectively).

#### GoKinD samples

One thousand two hundred and seventy-nine patients with 10 or more years of type 1 diabetes from the Genetics of Kidney Disease in Diabetes (GoKinD) were genotyped for p.R229Q. Four hundred and fifty-five of these subjects were "case" samples with end-stage renal disease, while the remaining 824 without end-stage renal disease served as "control" subjects. p.R229Q was found to have an overall allele frequency of 0.04 in the entire set of GoKinD samples. The allele frequency of the variant in "cases" with end-stage renal disease was 0.0297 and 0.039 in the control type 1 diabetics without nephropathy. There was no association between p.R229Q in the heterozygous state and proteinuria or end-stage renal disease in the case diabetics compared to normoalbuminuric control diabetics (chi-square 1.68, p = 0.20). In addition, the presence of a p.R229Q allele was not seen at greater frequency in subjects with elevated creatinine. p.R229Q Heterozygous individuals also did not have a higher incidence of abnormal creatinine (chi-square 1.314, p = 0.252) than individuals lacking this variant.

Renal disease is an established risk factor for cardiovascular disease [26]. p.R229Q was not seen at greater frequency in individuals with cardiovascular complications (chi-square 0.21 p = 0.65), hypertension (chi-square 1.31, p = 0.25), nor with subjects taking an angiotensin converting enzyme inhibitor (ACEi), or other antihypertensive medications (chi-square 0.002 and 0.99 with corresponding p values of 0.97 and 0.32).

We also found 3 samples homozygous for p.R229Q, but these were excluded from all our association studies. The homozygotes with p.R229Q had a 2.15 fold increase of developing proteinuria or ESRD but this did not reach statistical significance. This may be due to the very small number of subjects having this homozygous genotype (95% confidence interval 0.19–24.1), or it may be due to chance.

#### Australian endocrine diabetics

Four hundred and twenty-four patients with median diabetes duration of 20 years (range 3-to-58 years) enrolled through the Australian endocrine diabetes study were also studied. Sixty-seven had type 1 diabetes (51 with an urinary albumin excretion rate (AER) <20 μg/min, 10 with an AER between 20 μg/min and 200 μg/min, and the remaining 6 with an AER >20 μg/min) and the remaining 357 had type 2 diabetes (228 with an AER<20 μg/min, 83 with an AER between 20 μg/min and 200 μg/min, and the remaining 46 with an AER >200 μg/min). The p.R229Q variant had an allele frequency of 0.044 in the Australian endocrine diabetics. The allele frequency in cases with "nephropathy" (defined by an AER >20 μg/min) was 0.041 and 0.047 in the controls (subjects with an AER <20 μg/min) (Table 3). p R229Q did not associate with "nephropathy" (defined by an AER >20 μg/min) in the Australian endocrine diabetics (chi-square= 0.13, p = 0.72), micro or macroalbuminuria in type 1 diabetes (chi-square 1.39 and 0.5 with p values of 0.24 and 0.48 respectively), nor did it associate with micro or macroalbumiuria in type 2 diabetes (chi-square 0.44 and 0.04 with p values of 0.51 and 0.84 respectively).

#### No association between "diabetic nephropathy" and p.R229Q

When we pool the results from all 3 cohorts together and define a "case" diabetic as one with end-stage renal disease or abnormal proteinuria and a "control" as having no end-stage renal disease and normal urinary protein, no associations are present between p.R229Q and "diabetic nephropathy" (chi-square 0.67 and p value of 0.41).

## Discussion

We resequenced the coding sequence of *NPHS2 *in 371 individuals with FSGS and a median age of onset of disease of 25 years and found that 63 (or 17%) of the patients have at least one allele that alters the *NPHS2 *coding sequence, and of these, 12 (or 3.2% of the 371 screened) had these events in both *NPHS2 *alleles. Likely disease-causing mutations (homozygous or compound heterozygous) were identified in 4% (5/122) of the families and 2.8% (7/249) of the sporadics (excluding the homozygous and compound heterozygous events of p.R229Q and/or p.A242V).

We previously screened 30 multiplex families for *NPHS2 *mutations with adolescent or adult onset FSGS and identified 7 (23%) homozygous (n = 1) or compound heterozygous (n = 6) patients [6]. When these families are added to those screened here, we have identified likely disease-causing mutations in 8% of families (12 of 154 families). Our data show a non-trivial frequency of homozygous or compound heterozygous alleles in *NPHS2 *in late onset FSGS-affected individuals. Such genotypes are more frequent in patients with at least 1 other affected family member compared to sporadic FSGS patients (8% vs 2.8% respectively). These percentages are significantly smaller than most of the previous large studies which have focused on pediatric disease [15,17]. Several of the novel mutations identified in this study that both lead to amino acid substitutions and are present in the compound heterozygous state, with other previously known variants, are predicted to affect protein function of podocin using two SNP prediction algorithms; Sorting Intolerant From Tolerant (SIFT) and Polymorphism Phenotyping (PolyPhen). The p.R10T variant is predicted to be tolerated by SIFT and benign using Polyphen, p.V127W is predicted to be not tolerated by SIFT and possibly damaging by PolyPhen and p.L270F is predicted to be not tolerated by SIFT and possibly damaging using PolyPhen.

The finding of mutant *NPHS2 *alleles in sporadic cases confirms that disease that appears to be sporadic may in fact be inherited as a result of inheritance of mutant alleles from both parents, even in the absence of a positive family history of disease [27]. This may mean that homozygous or compound heterozygous events in other not-yet-identified genes that cause FSGS by recessive inheritance may underlie disease in these patients.

### The contribution of rare alleles to FSGS

We identified 51 patients (51/371, 14%) that had a heterozygous allele that altered the amino acid sequence without any other identified *NPHS2 *allele. The majority of these were the p.R229Q and p.A242V alleles (Table 1). Two of these alleles (p.T232I (c.694C>T) and p.L312V (c.934C>G)) are private non-conservative alleles that have not been identified in previous *NPHS2 *resequencing studies. The contribution of these rare alleles to disease is unclear. Neither was observed in any of the control alleles genotyped. The p.T232I allele was demonstrated in a subject with sporadic disease. The p.L312V allele was present in all affected siblings in a family with biopsy-confirmed FSGS. We cannot out rule the possibility of autosomal dominant disease in this family, as the father reportedly died from kidney impairment secondary to prostate cancer and the mother died of unknown causes. Interestingly, the p.T232I variant is predicted to be not tolerated by SIFT and possibly damaging by PolyPhen, and p.L327V is tolerated by SIFT and benign using PolyPhen. Rare heterozygous alleles in *NPHS2 *may possibly affect protein function, but these will need to be studied in cell culture to verify this.

Rare *NPHS2 *alleles have also been demonstrated in subjects with thin basement membrane nephropathy (TBMN). Tonna et al. 2003 identified a rare heterozygous variant (p.R224H (c.672G>A)) in only one patient with both TBMN and proteinuria, and like p.T232I the allele was not demonstrated in any control sample [28]. Without knowing the frequency with which non-proteinuric control groups carry single, rare, non-synonymous variants, it is difficult to know the clinical significance of such variants.

A small number of studies have attempted to show that heterozygous mutations in both the *NPHS2 *and *NPHS1 *genes in a single patient can cause FSGS [7,8,16]. It is unclear, however, what the frequency of such digenic events is in non-proteinuric (control) individuals. It is a straightforward hypothesis that digenic or perhaps even trigenic combinations of non-synonymous variants may occur in either of the *NPHS2*, *ACTN4*, *TRPC6*, *CD2AP*, or even *PLCE1 *genes in FSGS patients (especially those with sporadic disease). This may be in fact be the case in some of these patients with a single non-synonymous *NPHS2 *allele (especially if the allele is rare or is known to cause disease in the homozygous or compound heterozygous state), but confirming such a hypothesis will require extensive resequencing of many genes in FSGS cases as well as controls. It is however clear that 2.8% of patients with late onset, non-familial, FSGS have disease attributable to two mutant *NPHS2 *alleles.

### Common non-synonymous variants: the p.R229Q and p.A242V alleles

The most common non-synonymous changes we identified were the p.R229Q and p.A242V variants with population frequencies of 0.02 and 0.06, respectively, in the FSGS probands. We found one FSGS patient homozygous for p.R229Q, one homozygous for p.A242V, and one with compound heterozygosity for each of these alleles. Weber et al identified 3 families (2 of which are consanguineous) and 2 sporadic cases of FSGS with homozygosity for p.R229Q, [17]. This substitution is one of the most commonly reported *NPHS2 *alleles [6,8,14,15,20,21,25] with a greater frequency in Europeans (0.036) compared to the frequency in African Americans and Brazilians [6,17,25]. In another study, p.R229Q heterozygotes were found to have a 2.77 fold increased risk of developing microalbuminuria compared with controls [25], but it has been unclear whether this allele represents a genetic modifier for renal impairment in other diseases such as diabetic nephropathy.

To clarify whether these common non-synonymous variants cause or contribute to the development of albuminuria, we genotyped both of these alleles in cohorts of women from the Nurses' Health Study I and II. We observed that neither the p.R229Q nor the p.A242V allele were associated with increases in urine albumin/creatinine ratio in either the homozygous or heterozygous states. We found that p.A242V was present in an allele frequency of 0.034 in the Nurses' Health Study II, but in only 0.02 in the FSGS cohort. We note that this frequency in the control group (NHSII) is higher than that previously reported in control groups of similar ethnicity [29]. In the FSGS sample set, this variant was present in a higher frequency in persons of African descent compared to other ethnicities (chi-square with Yates correction 9.79, p = 0.00057), consistent with other studies [17]). It is not clear how the p.R229Q allele causes FSGS in the compound heterozygote state when inherited together with a second mutant allele [6,8,14-17,19,22], but not in the homozygous state, but this study confirms earlier suspicion that p.R229Q causes disease only in conjunction with a second more detrimental allele [6]. An earlier study reported that p.R229Q associates with microalbuminuric events in the population [25]. However, in the present study, we saw no association of the p.R229Q allele with albuminuria in either the Nurses' Health Study I nor in the diabetic populations analyzed. Further, the p.R229Q and p.A242V alleles in the homozygous state do not cause FSGS. The results of the genotyping of p.R229Q and p.A242V in the Nurses' Health Study reinforces the importance of evaluating potentially pathogenic variants in large populations.

### Pathogenicity of *NPHS2 *mutations

The biological effect of twelve mutant *NPHS2 *alleles (p.P118L, p.R138Q, p.R138X, p.D160G, p.R168H, p.R168C, p.R168S, p.V180M, p.R238S, DelLER (aa 237–239), p.V260E, and p.R291W) and two relatively common variants (p.P20L and p.G92C) have been studied in cell culture [30-32]. All but two of the mutants (p.V180M and p.R238S) fail to reach the plasma membrane in contrast to wild type podocin, whereas both the p.P20L and p.G92C variants do reach the plasma membrane. The lack of proper targeting of mutant *NPHS2 *to the plasma membrane has been shown to affect nephrin trafficking [30-32]. Other variants may have less avid binding to nephrin, as has been demonstrated in the case of p.R229Q [6]. However, lacking a systematic study of the frequency and biological effects of non-synonymous variants, it remains unclear which altered functions are meaningful markers of clinical pathogenicity.

## Conclusion

Mutations in *NPHS2 *are a rare cause of FSGS of late onset. Most (but not all) individuals with adult onset FSGS attributable to *NPHS2 *mutations have one p.R229Q allele. Our findings are consistent with other recent reports [9,10]. We have demonstrated that *NPHS2 *mutations contribute to FSGS in 8% of the familial cases and 2.8% of the sporadic cases analyzed here. Furthermore, the most commonly found polymorphisms demonstrated in *NPHS2 *resequencing, p.R229Q and p.A242V, do not appear to cause FSGS, nor associate with proteinuria in the homozygous or heterozygous state. In addition, p.R229Q heterozygous events do not associate with measures of kidney dysfunction in diabetic individuals, and thus p.R229Q is unlikely to represent a major genetic modifier for proteinuria.

## Competing interests

The authors declare that they have no competing interests.

## Authors' contributions

SJT, AN, SAW and KP performed genotyping studies. AU and JH coordinated the subject ascertainment. GBA, RJF, AK, BSK, GJ, JS performed clinical ascertainment of study subjects. SJT, KZ, GCC, and MRP performed statistical analyses. SJT and MRP drafted the manuscript. All authors read and approved the manuscript.

## Pre-publication history

The pre-publication history for this paper can be accessed here:


